# Strategic priorities for respiratory syncytial virus (RSV) vaccine development

**DOI:** 10.1016/j.vaccine.2012.11.106

**Published:** 2013-04-18

**Authors:** L.J. Anderson, P.R. Dormitzer, D.J. Nokes, R. Rappuoli, A. Roca, B.S. Graham

**Affiliations:** a Division of Pediatric Infectious Diseases, Emory University School of Medicine, 2015 Uppergate Dr, Atlanta, GA 30322, USA; b Novartis Vaccines and Diagnostics, 350 Massachusetts Ave., Cambridge, MA 02139, USA; c KEMRI-Wellcome Trust Research Programme, Centre for Geographic Medicine Research – Coast, PO Box 230, Kilifi 80108, Kenya; d School of Life Sciences, University of Warwick, Coventry CV4 7AL, United Kingdom; e Novartis Vaccines and Diagnostics Srl, Via Fiorentina 1, 53100 Siena, Italy; f MRC Unit, The Gambia, PO Box 273, Banjul, Gambia; g NIAID, NIH Vaccine Research Center Bldg. 40, Rm. 2502 40 Convent Drive MSC 3017, Bethesda, MD 20892, USA

**Keywords:** Respiratory syncytial virus, Vaccines, Global health priorities

## Abstract

Although RSV has been a high priority for vaccine development, efforts to develop a safe and effective vaccine have yet to lead to a licensed product. Clinical and epidemiologic features of RSV disease suggest there are at least 4 distinct target populations for vaccines, the RSV naïve young infant, the RSV naïve child ≥6 months of age, pregnant women (to provide passive protection to newborns), and the elderly. These target populations raise different safety and efficacy concerns and may require different vaccination strategies. The highest priority target population is the RSV naïve child. The occurrence of serious adverse events associated with the first vaccine candidate for young children, formalin inactivated RSV (FI-RSV), has focused vaccine development for the young RSV naïve child on live virus vaccines. Enhanced disease is not a concern for persons previously primed by a live virus infection. A variety of live-attenuated viruses have been developed with none yet achieving licensure. New live-attenuated RSV vaccines are being developed and evaluated that maybe sufficiently safe and efficacious to move to licensure. A variety of subunit vaccines are being developed and evaluated primarily for adults in whom enhanced disease is not a concern. An attenuated parainfluenza virus 3 vector expressing the RSV F protein was evaluated in RSV naïve children. Most of these candidate vaccines have used the RSV F protein in various vaccine platforms including virus-like particles, nanoparticles, formulated with adjuvants, and expressed by DNA or virus vectors. The other surface glycoprotein, the G protein, has also been used in candidate vaccines.

We now have tools to make and evaluate a wide range of promising vaccines. Costly clinical trials in the target population are needed to evaluate and select candidate vaccines for advancement to efficacy trials. Better data on RSV-associated mortality in developing countries, better estimates of the risk of long term sequelae such as wheezing after infection, better measures of protection in target populations, and data on the costs and benefits of vaccines for target populations are needed to support and justify funding this process. Addressing these challenges and needs should improve the efficiency and speed of achieving a safe and effective, licensed RSV vaccine.

## Highlights

► New technologies provide hope that an RSV vaccine can finally be achieved. ► There are opportunities to facilitate the chance of achieving an RSV vaccine. ► There are 4 potential target populations. Each has unique challenges and opportunities. ► Clinical trials of vaccines are needed to translate results in the lab to a human vaccine. ► Additional disease burden data is needed to appropriately prioritize RSV vaccine research.

## 1 Introduction

In preparation for The Decade of Vaccines Collaboration meeting in Sitges, Spain September 29–30 2011, we developed a case study for developing vaccines against respiratory syncytial virus (RSV) in support of the Research and Development Working Group. The purpose of this case study is to highlight challenges and opportunities for RSV vaccine development and identify priority activities that can facilitate vaccine development. This summary is based on the preparation for the meeting, discussions during the course of the meeting, and subsequent discussions among members of the RSV working group. Although no vaccine has yet been achieved, advances in molecular virology, immunology, and vaccinology, and a better understanding of pathology and pathogenesis, suggest that an RSV vaccine is within reach. This document identifies priority areas for future research and other activities to achieve the goal of a safe and effective RSV vaccine efficiently.

## 2 Background

Respiratory syncytial virus (RSV) is a major cause of lower respiratory tract infections in children worldwide, leading to an estimated 3 million annual hospitalizations and at least 66,000 deaths per year in children under 5 years of age. Rates of RSV hospitalizations are similar in developed and developing countries, at around 1% of children <1 year of age [1], [2], [3]. Mortality from RSV infection primarily occurs in developing countries, where the estimates are uncertain with the most recent ones being those noted above.

The burden of RSV globally has kept it a high priority for vaccine development. After nearly 50 years of attempts, there is still no licensed vaccine and there remain challenges to achieving a safe and effective licensed product. In this paper, we have outlined challenges to vaccine development and identified areas for future research or investigation that we believe are likely to improve the chance of achieving a safe and effective licensed vaccine. There are other sources including some recent reviews [4], [5], [6], [7], [8] to which readers should refer for more in depth discussions of the virus, its clinical and epidemiologic features, pathogenesis of disease, immunity to the virus, and vaccine platforms.

RSV is a negative sense, non-segmented RNA virus of the family *Paramyxovirdae* and subfamily *Pneumonvirinae*. Its genome has 10 genes that encode 11 proteins of which two surface glycoproteins, F and G, appear to be most important to inducing a protective immune response and one, the other, or potentially both will need to be included in an effective RSV vaccine. RSV infects more than 50% of children during the first year of life and the vast majority by 2–3 years of age [9], [10], [11]. Primary infections tend to cause the most severe disease but reinfections and severe disease occur throughout life [12]. The young infant and those with compromised cardiac, pulmonary, or immune systems as well as the elderly are at greatest risk of severe disease [13], [14]. The highest frequency of RSV-associated hospitalization in most studies is in children <6 months of age (peaking at 2–3 months of age) followed by older children (6–12 months of age), and there is substantial disease up to 5 years of age [1], [2], [15], [16]. Disease in older children appears to be especially important in developing countries [2], [10], [17], [18]. As illustrated by recent studies in Southern Africa, HIV-infected young children should be included in higher risk populations. In these studies HIV-infected children have at least a two-fold increase in rates of RSV lower respiratory tract hospitalization compared to HIV uninfected children[17], [19].

## 3 Target populations

The epidemiology and burden of RSV disease suggest that there are at least 4 distinct target populations for RSV vaccines: infants (<6 months of age) (highest risk of severe disease), children ≥6 months of age (both to prevent their disease and potential transmission to younger children and the elderly), pregnant women (to protect newborns both by transplacental transfer of antibodies and by blocking transmission), and the elderly (Table 1). Another vaccination strategy is to target those who can transmit the virus to high-risk persons or groups in the community. For example, vaccination might be used to block transmission from older siblings or other family members to infants or young children in the household; from health care workers to patients in the healthcare settings; or from children to the elderly.

**Table 1:** Key target populations for an RSV vaccine.

Target population	Key considerations	Primary vaccine approaches
0–6 months old infants	*Goal*: prevent serious complications of infection *Rationale*: highest rate of hospitalization *Challenges*: presence of maternal antibody; immature immune system; susceptibility to RSV disease; history of FI-RSV enhanced disease	(1) Live-attenuated RSV (2) Live chimeric virus vectors (3) Gene-based vectors (4) Potential for boosting sero-negative infants with subunit protein or particle-based vaccine after priming with live or gene-based vector vaccines

6–24 months old children	*Goal*: prevent serious complications of infection and reduce transmission to at-risk household contacts *Rationale*: ∼ 50% of childhood hospitalizations occur after 6 months of age; maternal antibody has waned; less susceptible to severe RSV disease and more mature immune system than younger children; potential to decrease transmission to others *Challenges*: clinical endpoint may be more difficult to achieve than in neonates; history of FI-RSV enhanced disease	(1) Gene-based vectors (2) Live-attenuated RSV (3) Live chimeric virus vectors (4) Potential for immunizing RSV-seropositive children with subunit protein or particle-based vaccine or boosting sero-negative children after priming with live or gene-based vector vaccines

Pregnant women or women of child-bearing age	*Goal*: increase passive antibody protection to fetus and prevent disease at most vulnerable age; block mother to infant transmission *Rationale*: high titer neutralizing antibody protects; can delay vaccination to older less vulnerable child with more mature immune system *Challenges*: having experienced multiple previous infections may limit response to vaccination; need for substantial increase in antibody levels to protect the infant; quantify the relationship between neutralizing antibody level and degree of protection	(1) Subunit protein with standard adjuvants (2) Particle including VLP with standard adjuvants

Adults >65 years	*Goal*: protect from serious complications of infection *Rationale*: substantial RSV-associated disease in elderly population *Challenges*: having experienced multiple previous infections may limit response to vaccination; need to improve on protection provided by natural infection; difficult to diagnose and lack of clear indicators of the severity of RSV disease	(1) Subunit protein with novel adjuvant (2) Particle including VLP with novel adjuvant (3) Gene-based vector with subunit protein or particle boost

The safety and efficacy concerns for each target population or vaccine strategy are different. Thus, there are likely opportunities for more than one type of vaccine, and choosing the target population best suited for a given vaccine or vaccine platform will be important to the vaccine's chances for success.

### 3.1 Infants (≤6 months of age)

The highest priority target population has been infants. Since several important transitions in RSV immunity occur at varying times in different infants, e.g. maturation of the infant immune system, waning of transferred maternal antibodies, and the first RSV infection, there is considerable heterogeneity in the immune status of infants and young children. Thus, the age of <6 months for this target population may need to be revised for different settings, for different vaccine platforms, and with new information on the duration of maternally acquired antibody and maturation of the immune system. Although RSV has been a high priority for vaccine development for this population for nearly 50 years, no vaccine is yet available. The first candidate vaccine, formalin-inactivated RSV (FI-RSV), was associated with enhanced disease and two deaths upon subsequent natural RSV infection [20], [21], [22], [23]. This occurred in children under 2 years of age but not in older children, possibly because among the older children prior natural infection established a safer immune response pattern prior to vaccination. These experiences directed development of RSV vaccines for the RSV-naïve child (mostly among infants) away from subunit and inactivated virus vaccines and toward live virus vaccines [4]. RSV infection in the infant, as well as young children, has also been linked to later reactive airway disease, though a causal relationship has not been established [24]. The infant presents challenges to vaccine development including an immature immune system [16] that may not respond well to a vaccine, possibility of recognized or unrecognized risk factors such as cardiac or lung disease or compromised immune systems, and elevated susceptibility to disease with live RSV infection. The lack of precise measures of disease severity makes assessing impact on disease in vaccinees, the goal of immunization for this population, more difficult.

### 3.2 Young children ≥6 months of age

Since many children >6 months of age will be RSV naïve, the issues and concerns regarding vaccines for young children >6 months of age are similar to those for the infant. The potential advantages of this target population include (1) having a more mature immune system and lower levels of maternal acquired antibody, making them more likely to have good responses to vaccines, and (2) likely being less susceptible to adverse respiratory events from infection with a live RSV vaccine. Though the preventable disease is less than that for the infant, it is probably sufficiently large to justify vaccination [1], [15].

### 3.3 Pregnant women

The primary goal for vaccinating pregnant women is to induce high levels of neutralizing antibodies that will be transferred to her fetus and protect the infant during the highest risk early months of life. The success of RSV immune prophylaxis [25] demonstrates that passive transfer of a sufficiently high titer of neutralizing antibody is likely to be protective. The potential for passive protection in the newborn is also supported by data on decreasing risk of disease in infants whose mothers had high titers of RSV neutralizing antibodies [26], [27], [28]. Another potential benefit of maternal immunization is preventing transmission from the mother to her infant. Live RSV vaccines have not been immunogenic in adults, and subunit vaccines are being considered for this target population. Since all adults have been infected by RSV multiple times, they are not considered at risk for vaccine-induced enhanced disease.

### 3.4 Elderly adults

Elderly adults have substantial burden of RSV disease, and this burden increases with underlying cardiac and pulmonary conditions [13]. A challenge to effective vaccination in the elderly population is immune senescence, which likely will make it more difficult to induce an effective immune response. Live-attenuated RSV vaccines have not been immunogenic in adults and subunit vaccines are being considered for this target population. Elderly persons most likely to benefit are often also those least able to respond to vaccination. The frequent presence of co-morbid conditions and lack of precise measures of disease severity make assessing impact on disease severity, the goal of vaccination in this population, more difficult.

### 3.5 Preventing RSV transmission

Another potential use for an RSV vaccine is preventing transmission to high-risk populations. Further study is needed to understand transmission in the community and the sources of spread to at-risk populations. Dynamic transmission models could be used to predict the potential impact of vaccination on transmission and help identify ways to study this effect [29]. Potential target populations to prevent transmission include health care workers caring for high risk patients; older children with infants and young children in their household; parents of young children; and children and workers in day care centers. To be successful, a vaccine will need to boost existing immunity to sufficiently high levels to prevent infection or decrease the risk of transmission if infection occurs. Of vaccines studied to date, the subunit vaccines appear most likely to achieve this goal. The tools to detect infection in vaccinees and in contacts are available and should meet the needs for assessing a vaccine's ability to prevent transmission, the goal of this vaccine.

## 4 RSV vaccines

Each type of vaccine and vaccine platform presents different challenges and opportunities. It has been difficult to achieve the right balance of safety and immunogenicity/efficacy for live attenuated RSV vaccines. These vaccines are, however, not considered a risk for inducing enhanced disease with subsequent RSV infection. Multiple attenuated viruses have been developed and evaluated as candidate live virus vaccines and not pursued. Others, including those noted below, are in various stages of development and evaluation. There are examples of both over- and under-attenuation in infants with under-attenuation being especially a concern for the very young infant, e.g. those 0–2 months [30], [31], [32]. A variety of attenuation strategies continue to be tried (e.g. deleting genes associated with immune response modulation or adding additional mutations associated with temperature sensitivity). Studies of the biology of infection and pathogenesis of disease in humans may suggest new and better ways to ensure the safety of a live virus vaccine and improve efficacy, but finding a single virus that will meet the diverse safety and efficacy needs of young children will remain a challenge. Clinical trials of new candidate live virus vaccines such as MEDI-559 (a live-attenuated RSV candidate vaccine) are continuing. Note that a live virus vaccine given intramuscularly at a relatively low dose was safe though not effective [33]. RSV given intramuscularly would have limited ability to replicate, and would likely not be immunogenic in persons with pre-existing RSV-specific antibody.

A variety of subunit RSV vaccines have also been developed [4]. The RSV F and G proteins are the only RSV proteins that induce neutralizing antibodies, best at inducing protective immunity in animals, and likely key components of a vaccine [8]. The F protein has been noted to induce higher levels of neutralizing antibodies and better protective immunity, be more conserved among RSV strains, and provide better cross-protection against different RSV strains than the G protein. The G glycoprotein is highly glycosylated and variable with the exception of the central region of the protein. Most candidate vaccines have focused on inducing antibodies against the RSV F protein. The success of passive antibody prophylaxis with an anti-F protein neutralizing monoclonal antibody [25], [34], [35] provides the proof-of-concept that a vaccine inducing sufficiently high levels of neutralizing antibodies to the F glycoprotein should prevent RSV disease.

Several approaches for simulating the antigen presentation that occurs during live RSV virus infection have been developed, leading to candidate vaccines such as virus vectors, gene-based vectors, replicons, and DNA plasmids [4]. Such candidate vaccines are designed to simulate the safe pattern of immune responses induced by live RSV infection, but without the risk of being insufficiently attenuated. These vaccines are intended to diminish the risk of enhanced disease in the RSV-naïve infant and young child that may occur when immunogens are processed as extracellular proteins or particles through MHC class II presentation pathways. In addition, these types of vaccines aim to avoid the problems of pre-existing immunity and potential for immune evasion and modulation associated with the live-attenuated RSV virus vaccine candidates. A bovine parainfluenza virus 3 expressing RSV-F (MEDI-534) has been studied in RSV-naïve infants and young children and not noted to cause enhanced disease [36].

A number of subunit vaccines that contain purified or expressed viral proteins have been developed and found to be safe in RSV-primed older children and adults [4]. The safety of these vaccines in older children and adults follows from the FI-RSV vaccine trials in which older children were not at risk from this vaccine and from animal model studies showing that prior live virus infection prevented FI-RSV enhanced disease [20], [21], [22], [23], [37]. Presumably, priming with live RSV infection patterned for a safe immune response and prevented the disease enhancing response. Though safe, protein subunit vaccines in older children and adults have demonstrated only modest immunogenicity as indicated by antibody responses [38], [39]. New F protein-based vaccines include those expressed as virus-like particles, incorporated into nanoparticles, or formulated with adjuvants with the hope of enhancing the protective immune response. One such vaccine, based on an insect cell-expressed F glycoprotein, is in early stage clinical trials. These vaccines have not been tested in RSV-naïve young children.

Although the clinical experience with passive antibody prophylaxis and protection in animals has focused vaccine development on the F protein, the contribution of other viral proteins to immunity and pathogenesis of disease should also be considered in designing future vaccines. For example, internal proteins like N, M, and M2-1 are rich in T cell epitopes and, if delivered by recombinant vectors, might improve vaccine-induced T cell mediated immunity [4], [40]. The G protein appears to play an important role in virus-induced host inflammatory responses that contribute to disease. G-specific antibodies might be used to bind G and block its ability to induce host inflammatory responses associated with disease [41]. Since both F and G induce neutralizing antibodies and protective immunity, a combination of F and G might improve the effectiveness of a vaccine. The small hydrophobic (SH) protein of RSV is thought to be a pentameric ion channel analogous to the M2 protein of influenza and is another vaccine antigen that should be considered. While these proteins are not targets for neutralizing antibody, other mechanisms like ADCC (antibody dependent cell-mediated cytotoxicity) might be elicited and contribute to protective immunity.

A major challenge to making progress in developing an RSV vaccine is translating promising results from in vitro and animal studies to humans. In vitro and animal model data have identified a number of promising candidate vaccines, but none has had a similar level of success in humans. At present, the only true indication of a candidate vaccine's safety and efficacy comes from clinical studies in the target population. Development of a more permissive and reliable animal model of disease enhancement could facilitate the safe testing of a greater variety of candidate vaccines in RSV-naïve infants.

The challenge of extrapolating from in vitro and animal studies to humans is compounded by the lack of good measures of RSV disease severity. The lack of precise measures of disease severity increases the size and cost of trials to assess the likelihood that a vaccine will be effective. Although preventing infection, if it can be achieved with a vaccine, would be a clean, easily measured end point, studies of passive immune prophylaxis with RSV-specific antibodies show that preventing disease can be achieved without preventing infection. Clinical endpoints, though imprecise, have and are likely to continue to be a key to defining efficacy. It may help to devise composite endpoints that include clinical and laboratory measures, e.g. biomarkers, of disease severity for inpatients and outpatients.

### 4.1 Challenges and opportunities

Although RSV has confounded efforts to develop a vaccine for nearly 50 years, the many new molecular, virology, and immunology tools now available should make it possible to achieve a safe, effective, licensed vaccine. Given the substantial public health benefit of such a vaccine, we should make every effort to use these tools most efficiently and effectively. The complexities of developing any new vaccine suggest a coordinated, collaborative approach that involves the various public, private and academic partners is most likely to succeed. We have identified barriers that, in most instances, apply to the development of any RSV vaccine and, therefore, should be addressed with a coordinated, collaborative approach through the concrete recommendations/actions noted below. In anticipation of successful development of an RSV vaccine, we have also identified potential barriers to implementation of vaccination programs.

### 4.2 The barriers to vaccine development

• The failures of RSV vaccines to date and the fact that natural infection provides limited protection from reinfection and disease indicate that the task of developing a safe and efficacious live virus vaccine will be difficult.
• Each vaccine target population presents distinct obstacles for RSV vaccine development.
  ∘ Infants <6 months of age have maternal antibody that provides partial protection but also interferes with the immunogenicity of live virus vaccines and with vectors to which the mother has antibodies. In addition, infants have an immature immune system that, among other deficiencies, has not fully developed the capacity for somatic mutation until 4–5 months of age and, thus, may respond to immunization with a more limited B cell repertoire. This very young, RSV-naïve population is considered at risk for vaccine-mediated disease enhancement.
  ∘ Many children 6 months to 2 years of age are still RSV-naïve and, therefore, are also at risk for vaccine-enhanced disease. Because they are past the peak age of hospitalization, vaccine developers have had less interest in developing vaccines for this group.
  ∘ Pregnant women, as well as older children and all adults will have pre-existing immunity making it necessary to improve on existing immunity and ensure that assays can accurately assess high-titered antibodies without missing responses because of assay saturation at high titers. For pregnant women, there is the concern of real or perceived risk to the fetus and potential vaccine liability for adverse fetal outcomes and the challenge of developing a program to vaccinate one group only to protect another group. Immunization of pregnant women may provide limited duration of infant protection, i.e. it is estimated that each additional month of protection to the infant will require a doubling of maternal antibody titers. In some populations the risk of severe disease following RSV infection does not significantly decline in the first 6 months of life, and maternal boosting may have less impact on disease. Maternal immunization may also decrease risk of RSV transmission from the mother to her child.
  ∘ The elderly have preexisting immunity that may make it difficult to boost and improve on existing immunity, and immuno-senescence may further decrease the effectiveness of vaccination. In addition, the frequent presence of underlying disease in elderly populations and short duration of viral shedding make it more difficult to document efficacy.
  ∘ Lack of data on chains of transmission in many settings makes developing this vaccination strategy difficult. It will require a sufficient boost in existing immunity to show a vaccine effect.
• There is not an ideal animal model for RSV vaccine evaluation. Studies of RSV in various animal models have provided important information on viral and host contributors to disease pathogenesis and response to candidate vaccines, but it is not clear how well this information applies to humans. Rodents (mice and cotton rats) and African green monkeys are semi-permissive to infection; there are not sufficient RSV-naïve chimpanzees available; and bovine RSV in cattle or pneumonia virus of mice are surrogate models that do not allow direct testing of the human vaccine products. There is not yet sufficient experience with human vaccine testing to conclude how well protection in animal models predicts protection in humans.
• Clinical studies of candidate vaccines in the target population are essential to determine which vaccines should be developed for licensure, but these studies are time consuming and expensive, and resources for these studies are limited. The ability to measure the impact of vaccine on disease is problematic in all target populations. Evaluating the impact of the vaccine on clinical endpoints has poor specificity as clinical presentations of RSV infections overlap with a range of other viral infections as well as asthma. Laboratory diagnosis of RSV infection is easiest in the infant and young child because they have higher titers of virus in respiratory secretions that are easier to detect. In adults the titers of virus are lower, and infection is only reliably detected by a rise in antibody between acute and convalescent serum samples or by sensitive RT-PCR assays. A decrease in the severity of disease is the most likely indication that a vaccine is effective. Clinical and laboratory measures of disease severity are imprecise at all ages. Imprecise measures of disease outcome compromise measures of vaccine efficacy and result in the need for larger and more costly studies.
• Since RSV-primed children and adults are not susceptible to enhanced disease, lack of enhanced disease in RSV-primed persons does not predict lack of enhanced disease in the RSV-naïve child, making it difficult to develop safety data to support testing novel RSV vaccines based on non-live virus platforms in the primary target population (infants <6 months).
• The clinical manifestations of RSV include wheezing and bronchiolitis similar to asthma. Because of that, it has been suggested that RSV may induce an inflammatory response in the host that significantly contributes to disease caused by the virus. If that is the case, there are some concerns that replication-competent vaccines may induce similar responses that may result in subsequent airway disease. The risk of vaccine-induced aberrant immune or inflammatory responses may be specific to certain groups, e.g. asthmatics, and suggests the need to evaluate vaccines in various well-characterized subpopulations.
• Children in developing countries present unique challenges, including higher frequency of pre-existing HIV infection and parasitic disease, higher rates of bacterial co-infection, and economic and logistical barriers to immunization.
• The lack of data on RSV-associated mortality, predisposition to subsequent severe respiratory disease in the short term (one study only [42]) and long term sequelae (risk of subsequent wheezing) has hindered accurate assessment of the costs and benefits of RSV vaccines and prioritization of vaccines for different target populations. Lack of good disease burden data is especially problematic in developing countries where severe cases and deaths concentrate, but also applies to children anywhere. Also problematic is the lack of good data on the risk of later reactive airway disease or asthma, the burden of disease in children >6 months of age, and the risk of older children transmitting virus to others at high risk of disease such as neonates and the elderly.
• There are limited resources to implement vaccination programs, and existing commitments for introduction of *Haemophilus influenza* type b, *Streptococcus pneumoniae* and rotavirus vaccines in developing countries may make it more difficult to introduce and use other vaccines, such as those for RSV.
• The long history of failed vaccine candidates for RSV raises commercial risks. However, the undeniable medical need and substantial predicted market support commercial investment. Immunization during pregnancy raises liability concerns that could dampen commercial interest in this approach.
• With any vaccine, there is the potential to confuse chance occurrence of disease with true vaccine-related adverse events. Data on backgrounds rates of disease and illness that might be considered possible adverse reactions to an RSV vaccine, such as sudden infant death syndrome (SIDS), will make it possible to assess the likelihood that adverse events are, or are not, related to the vaccination.

### 4.3 Concrete actions/recommendations for vaccine development

We feel the following types of research or other activities can address some of the most important challenges and needs noted above and by doing so can substantially improve the speed and probability of success in RSV vaccine development. The following recommendations are prioritized by magnitude of impact and likelihood of success in the next 3–5 years.

#### 4.3.1 Scientific recommendations: vaccine related

• Perform clinical trials with different types of candidate vaccines in different target populations. Clinical trials provide a unique and important opportunity to learn about RSV immunity and disease pathogenesis and provide the means to improve future candidate vaccines in the same or different target populations. Maximizing the information gained on response to vaccine, protective immunity, and disease pathogenesis for all trials should be a high priority.
• Establish precise measures of disease severity to use in assessing the impact of vaccines on disease for each potential target population. Development of clear, precise indicators of disease severity (e.g. clinical signs and symptoms, virology measures, immune response measures, biomarkers, etc.) will improve the efficiency and decrease the cost of vaccine trials and will likely increase the number and variety of types of vaccine evaluated in clinical trials.
• Define the protective and disease enhancing immune responses associated with natural infection and infection after vaccination, including correlates of protection in humans. This could include RSV challenge studies in adults to achieve a deeper understanding of immunity against reinfection and of factors associated with boosting existing immunity and achieving durable RSV protective immunity and to identify virus and host factors that contribute to pathogenesis of RSV disease. These studies would inform vaccine design and evaluation.
• Develop animal models and in vitro tissue culture systems that more reliably predict safety and efficacy of vaccines in humans.

#### 4.3.2 Scientific recommendations: epidemiology related

• Determine rates of RSV-associated mortality in developing countries.
• Determine the contribution of RSV infection in the infant and young child to later reactive airway disease.
• Model the costs and benefits of vaccinating various target populations using different vaccine strategies.
• Define chains of transmission to populations at risk from RSV infection.

#### 4.3.3 Commercial recommendations

• Because large and costly clinical studies will be required to determine which candidate vaccines to advance to licensure, new models for public, non-profit, and industry collaborations and public-private partnerships are needed to improve the efficiency and decrease the cost of trials and provide mechanisms to share information gleaned from clinical trials.
• Seek novel strategies, such as legislative solutions for no-fault compensation, to mitigate the liability associated with vaccination during pregnancy, and formulate guidance on the types of data and studies to assess the safety of protein-based and other inactivated virus vaccines in RSV-naïve infants and young children.
• Develop data on rates in the general population of rare adverse events and disease syndromes that might be confused with vaccine-induced adverse events.

#### 4.3.4 Programmatic recommendations

• Develop good surveillance and disease burden data for each potential affected population in different settings to guide resource allocation decisions.
• Develop educational tools for patients, clinicians, public health and government leaders about the RSV– disease burden for each affected population and setting, and articulate the costs and potential benefits of vaccination.
• Develop transmission models to demonstrate how immunization of one target population may protect others (e.g. immunization of young children protecting neonates and the elderly or immunization of pregnant women protecting neonates).

## 5 Summary

The tools that should allow us to develop a safe and effective RSV vaccine are available and our challenge is to use them wisely. We believe the *concrete actions/recommendations for vaccine development* noted above can help researchers, funding agencies, and industry focus their efforts and resources most efficiently and effectively.

## Conflicts of interest

Dr. Anderson has done paid consultancies on RSV vaccines and therapeutics for MedImmune, Inc. and Novartis Vaccines and Diagnostics; is a co-inventor on patents related to RSV anti-G protein antibody treatment and vaccines; and is doing animal studies on RSV anti-G protein treatment of RSV infection funded in part by Trellis, Inc.

Drs. Rino Rappuoli and Philip Dormitzer are employed by Novartis Vaccines and Diagnostics. Dr. Dormitzer has received expense reimbursements related to participation in scientific conferences and advisory boards by the Harvard-Armenise Foundation, BARDA, NIH, St. Judes Medical Center and PATH.

Dr. James Nokes is on an advisory panel for GSK and has a proposal pending to GSK to model RSV vaccines.

Dr. Graham is named on vaccine-related patents involving RSV antigen design and delivery approaches and involved with a CRADA (cooperative research and development agreement) with Genvec to develop recombinant adenovirus vector vaccines for RSV.

Dr. Roca reports no conflicts of interest.
